# Efficacy and safety of insulin efsitora in type 2 diabetes: a meta-analysis of randomized controlled trials

**DOI:** 10.3389/fendo.2025.1608458

**Published:** 2025-12-16

**Authors:** Yang Liu, Dingtao Chen, Ke He, Jichao Wei, Miao Liang, Li Han, Jingxin Li, Xuejing Li, Xianying Wang

**Affiliations:** 1 Department of Pharmacy, Hebei Medical University Third Hospital, Shijiazhuang, China; 2 School of Basic Medical Sciences, Hebei Medical University, Shijiazhuang, China; 3 Department of Clinical Pharmacy, The Fourth Hospital of Shijiazhuang/Shijiazhuang Obstetrics and Gynecology Hospital, Shijiazhuang, China; 4 Department of Pharmacy, Hebei Chest Hospital, Shijiazhuang, China

**Keywords:** insulin efsitora, insulin degludec, type 2 diabetes, meta-analysis, randomized controlled trials

## Abstract

**Objective:**

This meta-analysis aimed to evaluate the efficacy and safety profiles of insulin efsitora in the treatment of type 2 diabetes (T2D).

**Methods:**

We conducted a comprehensive systematic search across PubMed, the Cochrane Library, and Embase from database inception through September 11, 2025. The study included randomized controlled trials (RCTs) that directly compared insulin efsitora with once daily basal insulin in T2D patients. Primary outcomes assessed were changes in hemoglobin A1c (HbA1c) and body weight. Methodological quality and risk of bias were evaluated using the Cochrane Quality Assessment Tool. Data synthesis was performed using random-effects models to calculate risk ratios (RR) and mean differences (MD).

**Results:**

The meta-analysis incorporated six RCTs involving 4116 participants. Our findings revealed no statistically significant difference in HbA1c reduction between insulin efsitora and once daily basal insulins (MD: -0.04%; 95% CI: -0.10% to 0.02%; p = 0.78). Other outcomes, including change in body weight, body mass index changes, proportion of patients achieving HbA1c < 7%, change in fasting plasma glucose, and various hypoglycemia events (level 1, level 2, and level 3), as well as adverse events and serious adverse events, showed comparable results between the two treatments. Notably, insulin efsitora demonstrated superior performance in total daily insulin dose and time in range (70-180 mg/dL).

**Conclusions:**

Insulin efsitora demonstrates comparable efficacy and safety to once daily basal insulins in the management of T2D. However, given the limited number of RCTs available in the current evidence base, further large-scale clinical trials are warranted to validate these findings and establish more definitive conclusions.

## 1 Introduction

In the 21st century, type 2 diabetes (T2D) poses a significant and growing public health burden in the world. Approximately 536.6 million adults worldwide were affected by diabetes in 2021, a number projected to rise to 783.2 million by 2045 (1). T2D is characterized by impaired insulin secretion and reduced peripheral insulin sensitivity, leading to chronic hyperglycemia (2, 3). T2D management centers on lifestyle modifications and pharmacological interventions (4, 5). As the disease progresses, basal insulin is typically introduced when patients fail to achieve glycemic targets with oral antidiabetic agents or present with severely elevated HbA1c levels (5). Insulin efsitora stands out as a fusion protein combining a single-chain insulin variant with a human IgG Fc domain, conferring an extended half-life of approximately 17 days (408 hours) and enabling once-weekly dosing (6). By minimizing dosing frequency, insulin efsitora may have the potential to mitigate patient reluctance toward insulin therapy and reduce glycemic variability.

Several randomized controlled trials (RCTs) and one meta-analysis have evaluated insulin efsitora (7–10). However, the previous meta-analysis by Dutta et al. compared insulin efsitora with once-daily basal insulins without focusing exclusively on T2D patients. Conventional daily basal insulin, though a standard T2D treatment for patients failing oral agents or with severely elevated HbA1c, poses significant clinical challenges: high treatment burden, cost concerns, reduced flexibility, and reliance on others for administration (11). T2D management is evolving toward more convenient, patient-friendly regimens, and insulin efsitora represents a key innovation in this direction (12). To address this gap, it is imperative to systematically assess the efficacy and safety of once-weekly insulin efsitora versus once daily basal insulin specifically in T2D populations to inform better clinical practice decisions by healthcare professionals.

## 2 Materials and methods

This systematic review and meta-analysis was conducted in accordance with the Preferred Reporting Items for Systematic Reviews and Meta-Analyses (PRISMA) guidelines (13, 14). The study protocol was prospectively registered on PROSPERO (Registration ID: CRD42024607006) to ensure transparency and minimize bias.

### 2.1 Search strategy and selection criteria

We performed a systematic search of three major electronic databases (PubMed, EMBASE, and Cochrane Library) from their inception until September 11, 2025. Additionally, we identified registered studies through ClinicalTrials.gov. The search strategy incorporated a combination of Medical Subject Headings (MeSH) terms and keywords. The search terms utilized for the literature review were as follows: “diabetes mellitus [Mesh] OR diabetes [Title] OR T2DM [Title] OR DM [Title] OR T2D [Title] OR diabetes mellitus [Title] OR diabetes mellitus type 2 [Title] OR type 2 [Title]” AND “insulin efsitora [Title] OR basal insulin Fc [Title] OR LY3209590 [Title] OR BIF [Title]”.

Studies were included if they met the following criteria: Design: RCTs; Population: Patients with T2D; Intervention: Insulin efsitora; Comparator: once daily basal insulin; Outcomes: Efficacy outcomes: change in HbA1c, change in body weight, body mass index (BMI) changes, change in time in range (TIR) (70–180 mg/dL), percentage of patients achieving HbA1c < 7%, fasting plasma glucose (FPG), and total daily insulin dose. Safety outcomes: Level 1 hypoglycemia, level 2 hypoglycemia, level 3 hypoglycemia, adverse events (AEs), and serious adverse events (SAEs). We excluded case reports, cohort studies, conference abstracts, letters, comments, duplicate studies, and RCTs with insufficient or unusable data. We also excluded the RCTs which involved minors, gestational diabetes or other special diabetic groups. Two independent reviewers conducted the study selection. Titles and abstracts were screened to exclude irrelevant studies. Potentially eligible articles underwent full-text assessment based on predefined inclusion and exclusion criteria. Any discrepancies between reviewers were resolved through discussion with a third researcher to reach consensus.

### 2.2 Data extraction and risk of bias assessment

Two investigators independently extracted data from the included studies using a pre-designed, standardized Excel form. The extracted data encompassed: study and patient characteristics: trial name, country, interventions, sample size, age, sex distribution, body weight, BMI, baseline HbA1c, diabetes duration, and treatment duration; efficacy outcomes: change in HbA1c, change in body weight, change in TIR (70-180 mg/dL), proportion of patients achieving HbA1c < 7%, FPG, and total daily insulin dose; safety outcomes: level 1 hypoglycemia, level 2 hypoglycemia, level 3 hypoglycemia, AEs, and SAEs. For studies where mean differences (MD) and standard deviations (SD) were not directly reported, these values were estimated using established methods by Hozo et al. and Wan et al. (15, 16). Two reviewers independently evaluated the methodological quality of the included RCTs using the Cochrane Risk of Bias Tool (17). Discrepancies were resolved through discussion with a third reviewer. The following domains were assessed and categorized as low risk, high risk, or unclear risk: random sequence generation, allocation concealment, blinding of participants and personnel, blinding of outcome assessors, incomplete outcome data, selective reporting, and other potential biases. Risk-of-bias graphs were generated using RevMan 5.4 software to visually summarize the findings.

### 2.3 Statistical analysis

This meta-analysis employed RevMan 5.4 and Stata 16.0 software for statistical computations. Effect sizes were calculated as risk ratios (RR) with 95% confidence intervals (CIs) for dichotomous outcomes, and mean differences (MD) with corresponding 95% CIs for continuous variables. Between-study heterogeneity was assessed using both the I² statistic and Cochrane’s Q test, with predefined thresholds of p ≤ 0.10 or I² > 50% indicating substantial heterogeneity. A random-effects model was applied for pooled analyses to account for variability across studies. Publication bias was evaluated through visual inspection of funnel plot asymmetry. Meta regression analysis and sensitivity analyses were additionally performed to assess the robustness of findings.

## 3 Results

### 3.1 Database search results and quality assessment

**Figure 1:**
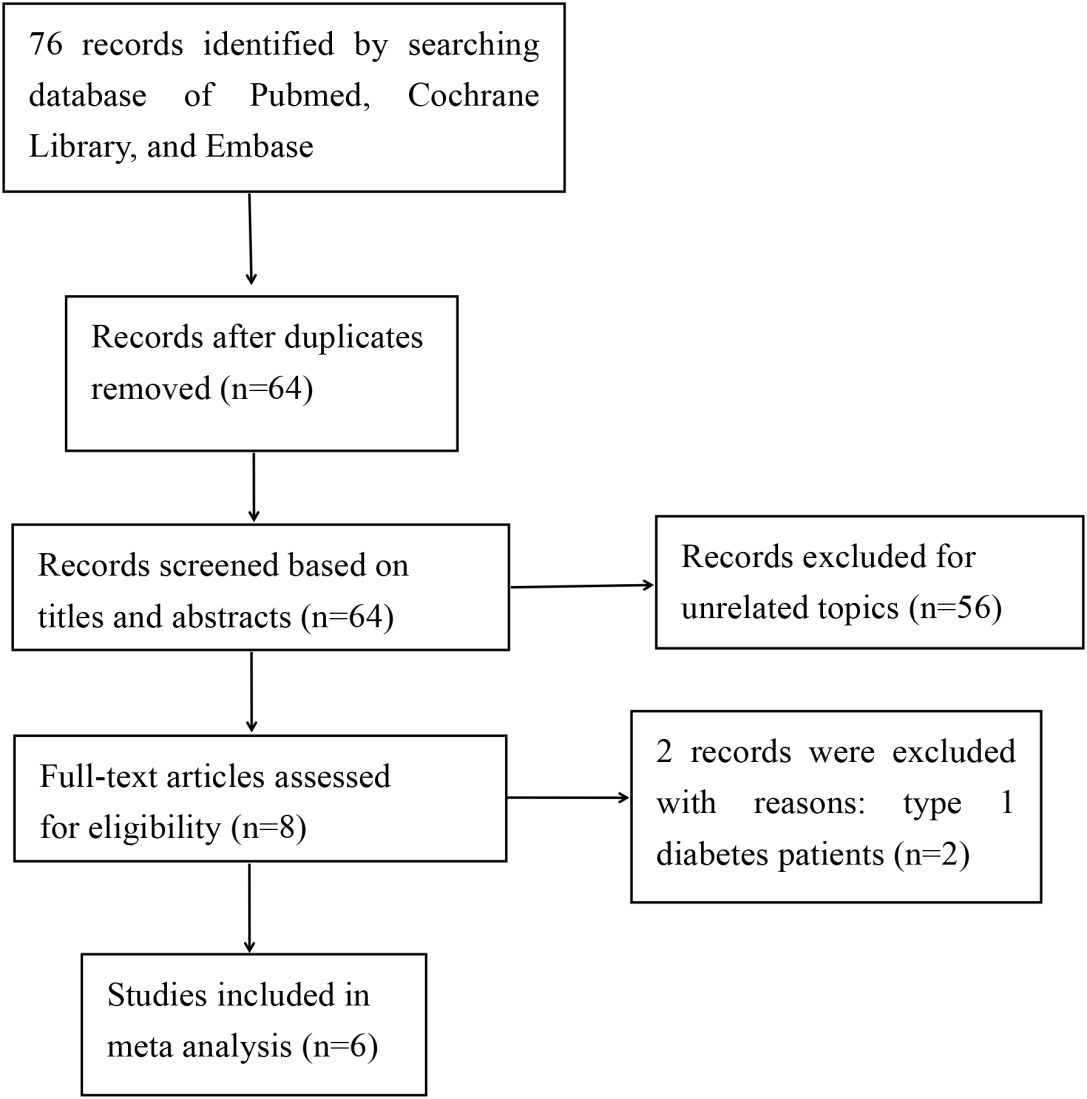
The selection process of included studies.

**Figure 2:**
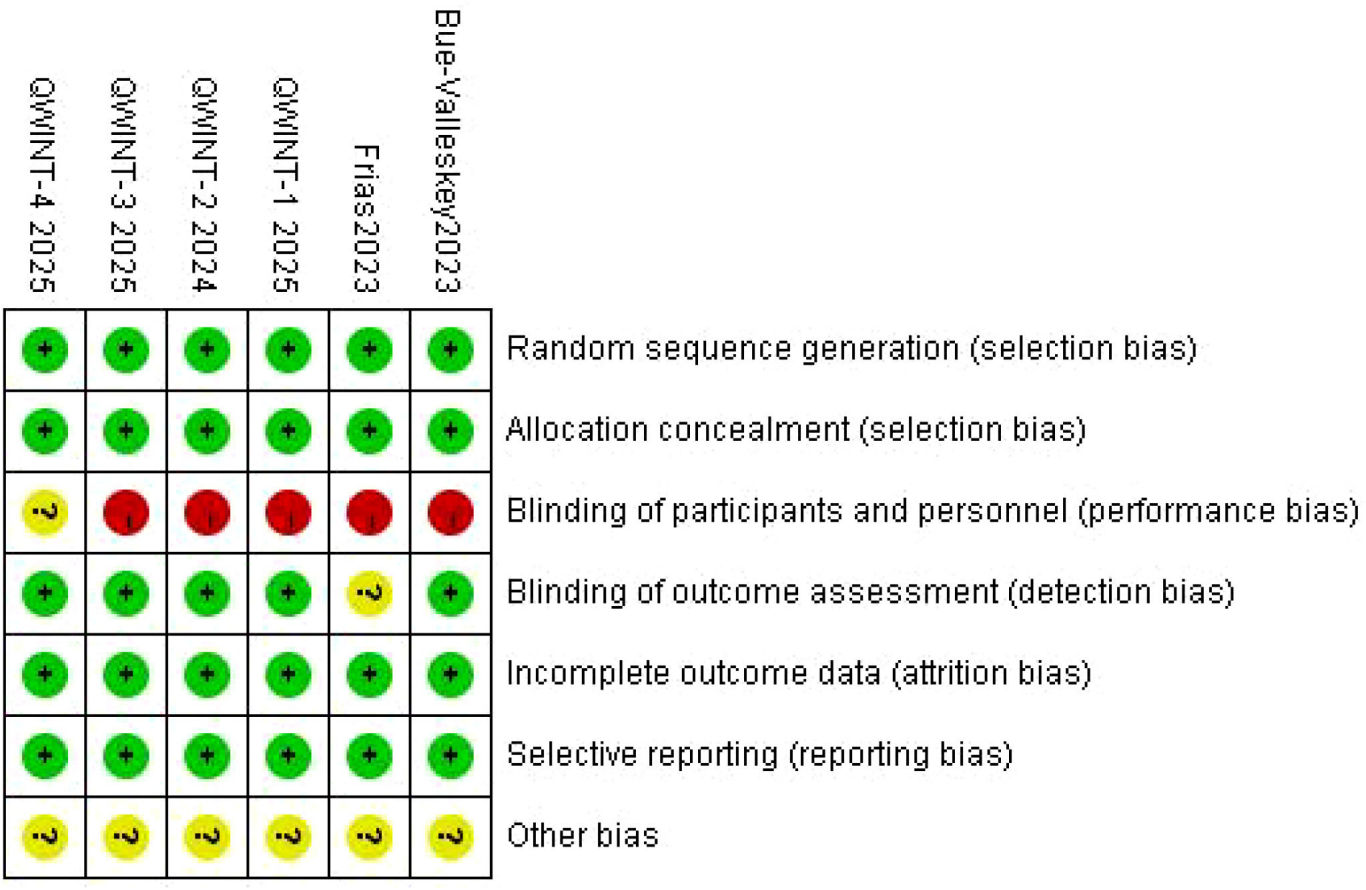
Quality assessment of included studies.

A systematic search across three electronic databases and clinical trial registries identified 76 potentially eligible records. After removing 12 duplicates, 64 unique studies underwent title and abstract screening, which excluded 56 records due to irrelevance or failure to meet inclusion criteria. The remaining 8 articles were assessed in full-text, ultimately yielding 6 studies for qualitative synthesis (7–9, 18–20). **Figure 1** illustrates the PRISMA-compliant flow diagram detailing study selection. **Table 1** summarizes the key features of the included trials, all published between 2023 and 2025. Sample sizes ranged from 278 to 986 participants, with follow-up durations spanning 26-78 weeks. Baseline characteristics were comparable between intervention groups: mean BMI values across studies were 29.6-32.5 kg/m^2^, with diabetes durations of 9.72-16.9 years. All trials adopted a parallel-group design and were classified as high-quality based on Cochrane Risk of Bias criteria (**Figure 2**). The efficacy and safety profiles were summarized in **Table 2**.

**Table 1:** Characteristics of the included studies and patients.

Trial name	Country	Study arms	Patients	Age (years)	Male, n (%)	Body weight (kg)	Body-mass index (kg/m^2^)	HbA1c (%)	Duration of diabetes (years)	Treatment duration (weeks)
QWINT-2 2024	Brazil, Canada, China, et al	Efsitora	466	57.6 ± 10.6	281 (60.3)	86.83 ± 20.53	30.44 ± 5.85	8.21 ± 0.96	11.78 ± 7.54	52
		Degludec	462	57.3 ± 11.0	265 (57.4)	86.12 ± 18.93	30.72 ± 5.90	8.23 ± 0.96	11.42 ± 6.97	
Bue-Valleskey2023	Argentina, Germany, Poland, et al	Efsitora	143	57.3 ± 9.7	76(53.1)	91.0 ± 20.8	32.3 ± 5.4	8.1 ± 0.8	10.4 ± 6.8	26
		Degludec	135	59.4 ± 9.1	76(56.3)	90.6 ± 19.6	31.6 ± 5.5	8.0 ± 0.8	9.7 ± 6.0	
Frias2023	USA, Puerto Rico, and Mexico	Efsitora	267	59.9 ± 10.6	130(49)	89.4 ± 19.2	32.5 ± 5.9	8.1 ± 0.9	14.6 ± 8.8	32
		Degludec	132	60.8 ± 10.0	67(51)	87.1 ± 20.7	31.8 ± 5.7	8.1 ± 0.9	15.1 ± 8.0	
QWINT-1 2025	Argentina, Mexico, and the United States	Efsitora	397	56.4 ± 10.0	203(51.1)	89.3 ± 19.2	32.5 ± 5.8	8.20 ± 0.91	9.2 ± 6.6	52
		Glargine	398	56.2 ± 9.7	195(49.0)	85.5 ± 19.7	31.3 ± 6.1	8.27 ± 1.07	9.6 ± 6.9	
QWINT-3 2025	Argentina, Hungary, Japan, et al	Efsitora	655	62	376(57)	83.6	29.6	7.7	14.5	78
		Degludec	331	62	179(54)	84.3	29.8	7.7	14.4	
QWINT-4 2025	Argentina, Germany, India, et al	Efsitora	365	58.3 ± 10.5	172(47)	87.8 ± 19.9	31.85 ± 5.48	8.36 ± 0.78	16.6 ± 8.8	26
		Glargine	365	59.4 ± 10.5	189(52)	88.4 ± 19.7	31.84 ± 5.48	8.36 ± 0.80	16.9 ± 9.0	

**Table 2:** Summary of the efficacy and safety profiles of insulin efsitora for T2D patients.

Outcomes	Included trials	Included patients	Statistical method	RR/MD (95%CI)
Change in HbA1c	6	3979	MD (M-H, Random, 95% CI)	-0.04 [-0.10, 0.02]
Change in body weight	5	3742	MD (M-H, Random, 95% CI)	0.05 [-0.32, 0.42]
Change in time in range (70-180 mg/dL)	3	1590	MD (M-H, Random, 95% CI)	0.52 [0.05, 1.00]
Percentage of patients achieving HbA1c < 7%	3	1987	RR (M-H, Random, 95% CI)	1.02 [0.93, 1.12]
Change in FPG	6	3960	MD (M-H, Random, 95% CI)	0.14 [-0.14, 0.42]
Total daily insulin dose	5	3358	MD (M-H, Random, 95% CI)	-4.49 [-8.15, -0.83]
Hypoglycemia alert (level 1)	6	4115	RR (M-H, Random, 95% CI)	1.04 [0.97, 1.11]
Clinically significant hypoglycemia (level 2)	5	3385	RR (M-H, Random, 95% CI)	1.04 [0.87, 1.25]
Severe hypoglycemia (level 3)	6	4101	RR (M-H, Random, 95% CI)	0.97 [0.42, 2.24]
AEs	6	4115	RR (M-H, Random, 95% CI)	1.03 [0.96, 1.11]
SAEs	6	4115	RR (M-H, Random, 95% CI)	1.19 [0.97, 1.47]

### 3.2 Efficacy outcomes

**Figure 3:**
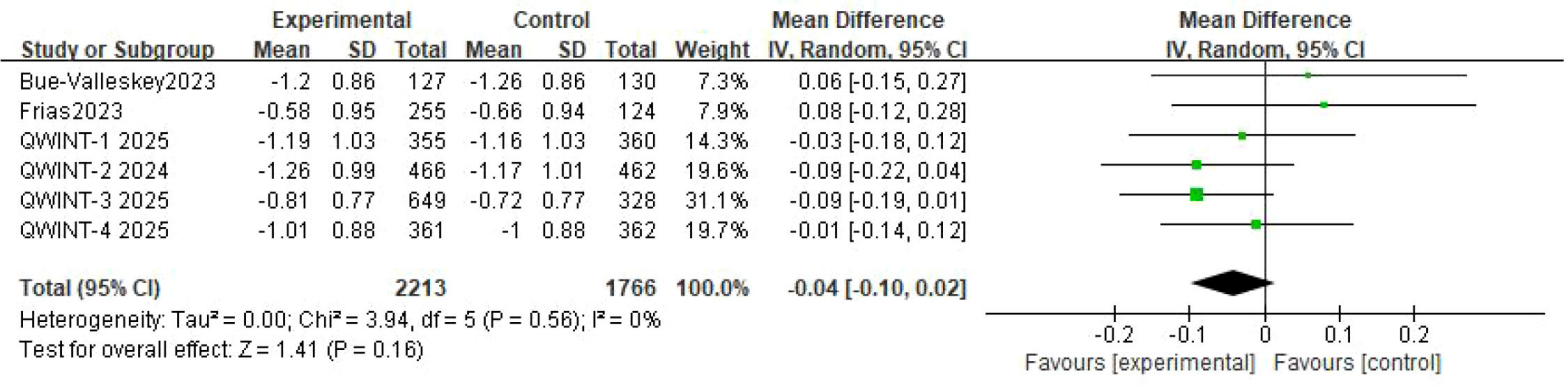
A forest plot illustrating the change in HbA1c from baseline in patients with T2D.

Six RCTs evaluated change in HbA1c in the overall T2D population. A pooled analysis of these studies showed no statistically significant difference in HbA1c reduction from baseline between insulin efsitora and once daily basal insulins (mean difference [MD] -0.04%; 95% confidence interval [CI]: -0.10% to 0.02%; p = 0.16). Heterogeneity was low (p = 0.56, I² = 0%) (**Figure 3**).

Five RCTs reported change in body weight in the overall T2D population. Meta-analysis of these studies found no significant difference between the two insulin groups (MD 0.05 kg; 95% CI: -0.32 kg to 0.42 kg; p = 0.78) (**Supplementary Figure S1**). Heterogeneity was high (p = 0.03, I²= 62%).

Data from two RCTs reporting BMI changes in the overall T2D population were pooled in a meta-analysis. This analysis yielded a non-significant MD of 0.04 kg/m² (95% CI: -0.07 to 0.15 kg/m²; p = 0.49) between the insulin regimens (**Supplementary Figure S2**). Heterogeneity was low (p = 0.73, I²= 0%).

TIR (70-180 mg/dL) data were available from three studies involving 1,590 participants. Pooled results showed insulin efsitora was superior to once daily basal insulin in TIR (MD 0.52%; 95% CI: 0.05% to 1.00%; p = 0.03) with no heterogeneity (p = 0.80, I² = 0%) (**Supplementary Figure S3**).

Three studies reported the percentage of patients achieving HbA1c <7%. As shown in **Supplementary Figure S4**, the rate was comparable between insulin efsitora and once daily basal insulin groups (relative risk [RR] 1.02, 95% CI: 0.93 to 1.12, p = 0.68) with moderate heterogeneity (I² = 36%, p = 0.21).

FPG changes were analyzed across six studies (n=3960). FPG levels were similar between insulin efsitora and once daily basal insulin groups (MD 0.14 mmol/L; 95% CI: -0.14 mmol/L to 0.42 mmol/L; p = 0.32) with moderate heterogeneity (p = 0.002, I² = 74%) (**Supplementary Figure S5**).

Total daily insulin dose data from five studies (n=3358) showed that patients in insulin efsitora group need smaller insulin dose than that in once daily basal insulin group (MD -4.49 U; 95% CI: -8.15 U to -0.83 U; p = 0.02) with moderate heterogeneity (p = 0.002, I² = 76%) (**Supplementary Figure S6**).

### 3.3 Safety outcomes

Level 1 hypoglycemia data from six studies (n=4115) showed no statistically significant difference between insulin efsitora and once daily basal insulin groups (RR 1.04, 95% CI: 0.97 to 1.11, p = 0.24) with high heterogeneity (I² = 65%, p = 0.01) (**Supplementary Figure S7**).

Level 2 hypoglycemia was evaluated in five studies (n=3385), with no significant difference between groups (RR 1.04, 95% CI: 0.87 to 1.25, p = 0.67) and high heterogeneity (I² = 65%, p = 0.02) (**Supplementary Figure S8**).

Six studies (n=4101) reported level 3 hypoglycemia, showing no significant difference between insulin efsitora and once daily basal insulins (RR 0.97, 95% CI: 0.42 to 2.24, p = 0.94) with no heterogeneity (I² = 0%, p = 0.41) (**Supplementary Figure S9**).

AEs data from six studies (n=4115) showed no significant difference between groups (RR 1.03, 95% CI: 0.96 to 1.11, p = 0.38) with low heterogeneity (I² = 56%, p = 0.04) (**Supplementary Figure S10**).

SAEs were reported in six studies (n=4115), with no significant difference between insulin efsitora and once daily basal insulins (RR 1.19, 95% CI: 0.97 to 1.47, p = 0.10) and no heterogeneity (I² = 0%, p = 0.71) (**Supplementary Figure S11**).

### 3.4 Meta-regression and sensitivity analysis

As presented in **Supplementary Table S1**, none of the covariates (age, male ratio, BMI, HbA1c, duration of diabetes) were identified as significant factors contributing to statistical heterogeneity. Moreover, as illustrated in **Supplementary Figures S12**-**Supplementary Figures S14**, our analysis consistently demonstrated uniform overall results regarding change in HbA1c, TIR (70-180 mg/dL), and SAEs, even after individually excluding each study included in the analysis. This consistency underscores the robustness and reliability of our findings.

## 4 Discussion

The current research indicates that insulin efsitora, administered once weekly, exhibits comparable efficacy and safety profiles to once-daily basal insulins when evaluated across the broader population of patients with T2D. Our study findings provide evidence that insulin efsitora performs similarly to once-daily basal insulins across several key metrics, including change in HbA1c levels, change in body weight, BMI changes, the proportion of patients achieving an HbA1c level below 7%, change in FPG, and the incidence of hypoglycemic events (categorized as level 1, level 2, and level 3), as well as AEs and SAEs. Notably, insulin efsitora demonstrated a superior capacity for TIR (70-180 mg/dL), and total daily insulin dosage compared to once-daily basal insulins. Furthermore, the outcomes derived from meta-regression analysis and sensitivity analysis reinforced the reliability and robustness of our meta-analysis conclusions.

Our study demonstrated that insulin efsitora provided a statistically significant, albeit modest, improvement in TIR compared to once-daily basal insulins (MD: 0.52%; 95% CI: 0.05%-1.00%; p=0.03). This finding offers practical support for the utility of TIR as a complementary metric to HbA1c, as it captures glycemic excursions that HbA1c alone may not reflect. Furthermore, when interpreted in the context of established international consensus (21–26) which links higher TIR to reduced complication risks, our results suggest that even incremental improvements in TIR, as seen with insulin efsitora, could potentially contribute to long-term clinical benefits.

Our meta-analysis showed that the mean total daily insulin dose in the efsitora group was 4.49 U lower than that in the once daily basal insulin group. Administered as a fixed-dose regimen, efsitora delivered glucose-lowering efficacy comparable to that of daily insulin while requiring fewer dose increases (9–11). This fixed-dose approach also reduced the need for multiple small dose adjustments-a common problem of daily basal insulin therapies. Such frequent adjustments can burden both clinicians and patients, potentially leading to therapeutic inertia and suboptimal glycemic control (27–29). For patients with T2D, efsitora’s simple, once-weekly fixed-dose regimen may simplify the initiation and management of insulin therapy for both parties, which aligns with observations for another once-weekly insulin, insulin icodec (30–34).

For patients with poor adherence to daily injections (e.g., elderly T2D patients with comorbidities and polypharmacy, or those reluctant to initiate insulin due to injection burden), insulin efsitora’s once-weekly dosing provides a more convenient alternative-addressing a major barrier to insulin initiation and potentially improving long-term treatment persistence (35, 36). The Diabetes Treatment Satisfaction Questionnaire (DTSQ) score is a well-established metric utilized to evaluate satisfaction levels with various innovative T2D therapies (37). Notably, two studies (38, 39) have indicated that, in comparison to once-daily basal insulin, once-weekly insulin administration results in a significantly higher DTSQ score, suggesting enhancements in daily living activities. QWINT-3 (19) concluded that efsitora-treated participants showed significantly higher levels of treatment satisfaction compared with degludec, as assessed by the DTSQ diabetes questionnaire. For patients, this translates to better integration of treatment into daily life (e.g., fewer disruptions to work/travel) and reduced psychological stress associated with frequent injections, ultimately promoting sustained engagement in diabetes care.

Research has revealed that intensive blood glucose control corresponds with a reduction of 10% in the risk of encountering various diabetes-related complications, encompassing heart attacks, stroke, amputation, and microvascular diseases (40). This meta-analysis substantiates that efsitora is effective in lowering HbA1c levels. Insulin efsitora’s comparable efficacy/safety to daily insulins, combined with its adherence advantage, suggests it may reduce long-term healthcare costs by lowering the incidence of complications caused by poor adherence to daily insulins (41, 42). Our evidence supports further investigation on insulin efsitora as an alternative option in patients with poor adherence.

Hypoglycemia is a significant concern for individuals living with T2D (43, 44). Considering the extended dosing interval and prolonged duration of action associated with insulin efsitora, there is potential for concerns regarding prolonged hypoglycemia, delayed recovery from hypoglycemic episodes, and recurrent hypoglycemia. However, our meta-analysis revealed that once-weekly insulin efsitora exhibits a favorable side-effect profile, with incidence rates of level 1, level 2, and level 3 hypoglycemic events comparable to those observed with once-daily basal insulins. Notably, insulin efsitora demonstrates a weekly peak-to-trough ratio of 1.14 (6), indicating a stable pharmacokinetic profile throughout the week, with only a 14% increase in insulin activity on the day of maximum observed concentration (6). This low peak-to-trough ratio may contribute to the favorable safety profile observed in our study.

This is the first systematic review and meta-analysis to comprehensively analyze the effectiveness and safety of once-weekly insulin efsitora when compared to once-daily insulins. Compared with previous meta-analysis (10), we compared insulin efsitora not only with insulin degludec but also with insulin glargine. We have added 3 newly published RCTs and made meta regression and sensitivity analysis compared with previously published meta-analysis. By systematically synthesizing 6 RCTs involving 4116 T2D patients, we provide the first dedicated evidence on the head-to-head comparison between insulin efsitora and once-daily basal insulins in T2D population. This enriches the evidence base for long-acting basal insulin therapy in T2D.

This study has limitations. Initially, it is worth noting that most of the RCTs incorporated into this research featured relatively brief follow-up periods, spanning 26 to 78 weeks. Consequently, the long-term outlook for patients receiving insulin efsitora treatment remains uncertain and necessitates ongoing monitoring. Secondly, the limited number of trials considered, which led to a comparatively small aggregate participant pool, may have implications for the robustness of the conclusions drawn. Additionally, substantial heterogeneity was observed in certain outcomes, attributed to variations in sample sizes and follow-up protocols across studies. Finally, we did not assess publication bias due to the limited number of RCTs included. As such, the findings should be approached with prudence.

## 5 Conclusions

Insulin efsitora demonstrates comparable efficacy and safety to once-daily basal insulins in the management of T2D.

However, given the limited number of RCTs available in the current evidence base, further large-scale clinical trials are warranted to validate these findings and establish more definitive conclusions.

## Data Availability

The original contributions presented in the study are included in the article/**Supplementary Material**. Further inquiries can be directed to the corresponding authors.
