# Supplementary material for: Efficacy and safety of insulin efsitora in type 2 diabetes: a meta-analysis of randomized controlled trials
Source: Front Endocrinol (Lausanne). 2025 Dec 16;16:1608458. doi: 10.3389/fendo.2025.1608458 (PMC12749861; doi:10.3389/fendo.2025.1608458)
Supplement: Supplementary file 1 [file SupplementaryFile1.doc]

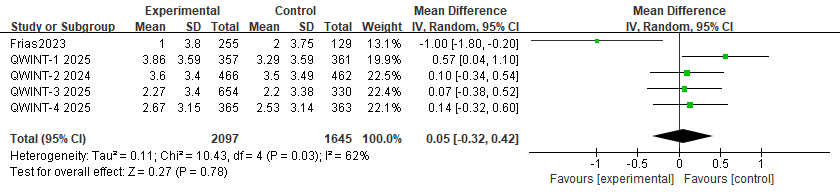


**Supplementary Figure 1 A forest plot illustrating the change in body weight from baseline in patients with T2D**


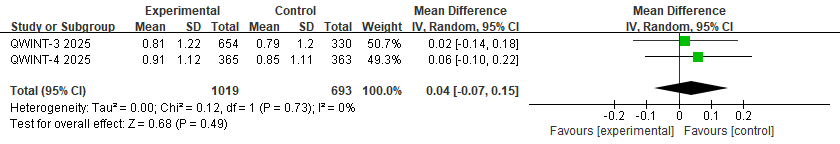


**Supplementary Figure 2 A forest plot illustrating the BMI changes from baseline in patients with T2D**


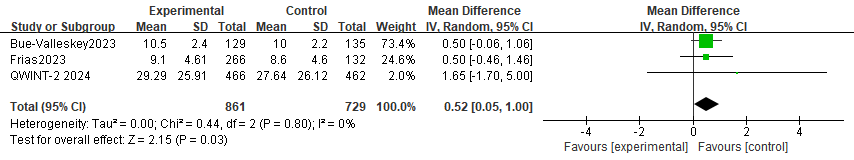


**Supplementary Figure 3 A forest plot illustrating the TIR (70-180 mg/dL) in patients with T2D**


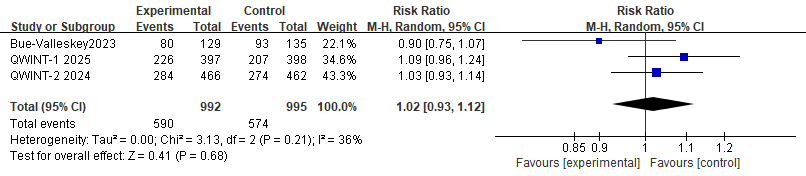


**Supplementary Figure 4 Effect of insulin efsitora on percentage of patients achieving HbA1c < 7.0% in patients with T2D**


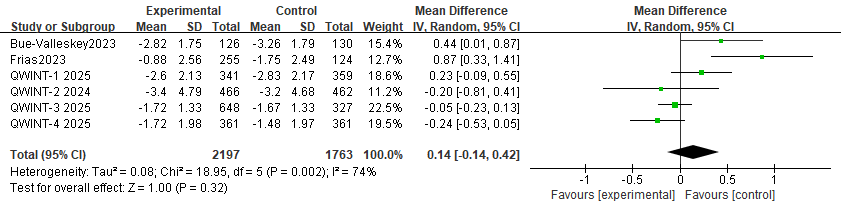


**Supplementary Figure 5 A forest plot illustrating the change in FPG from baseline in patients with T2D**


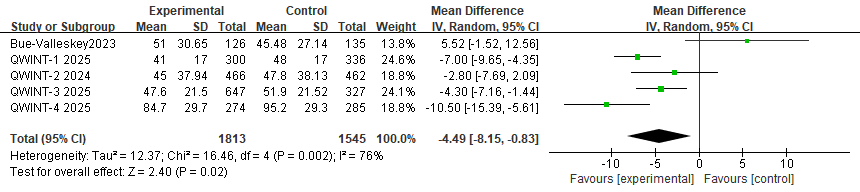


**Supplementary Figure 6 A forest plot illustrating the total daily insulin dose in patients with T2D**


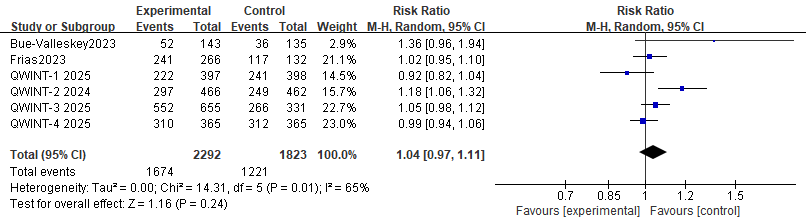


**Supplementary Figure 7 Effect of insulin efsitora on level 1 hypoglycemia in patients with T2D**


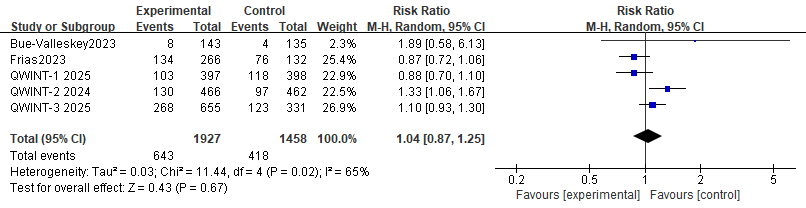


**Supplementary Figure 8 Effect of insulin efsitora on level 2 hypoglycemia in patients with T2D**


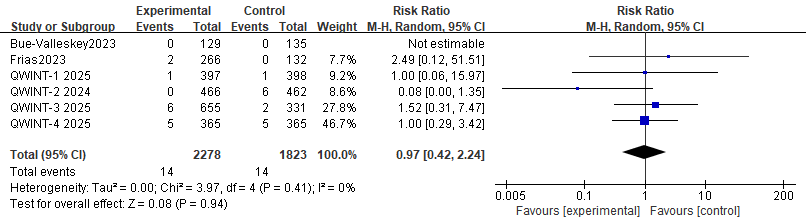


**Supplementary Figure 9 Effect of insulin efsitora on level 3 hypoglycemia in patients with T2D**


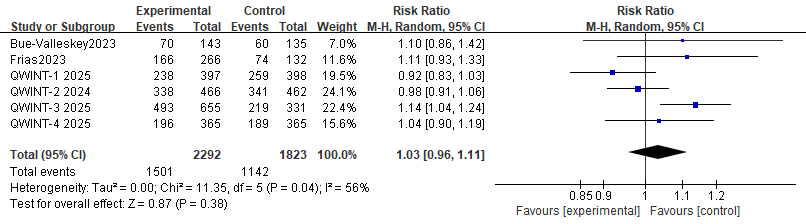


**Supplementary Figure 10 Effect of insulin efsitora on AEs in patients with T2D**
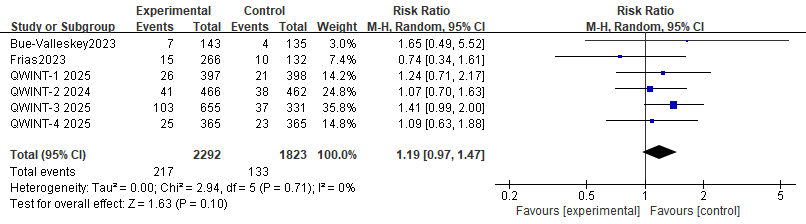


**Supplementary Figure 11 Effect of insulin efsitora on SAEs in patients with T2D**

**Supplementary Figure 12 Sensitivity analysis of change in HbA1c**

**Supplementary Figure 13 Sensitivity analysis of TIR (70-180 mg/dL)**

**Supplementary Figure 14 Sensitivity analysis of SAEs**

**Supplementary Table 1 Meta-regression on factors affecting change in HbA1c, change in time in range (70-180 mg/dL), SAEs**

| Outcome | Independent variable | Coefficient | 95% CI | | P-value |
| --- | --- | --- | --- | --- | --- |
| Change in HbA1c | Age (years) | -0.0067082 | -0.0452363 | 0.0318199 | 0.654 |
|  | Male (%) | -1.087741 | -3.2286 | 1.053118 | 0.231 |
|  | BMI (kg/m^2^) | 0.0484028 | -0.030873 | 0.1276785 | 0.165 |
|  | HbA1c (%) | 0.0944286 | -0.2162962 | 0.4051535 | 0.446 |
|  | Duration of diabetes (years) | 0.0005579 | -0.0312442 | 0.03236 | 0.963 |
| Change in time in range (70-180 mg/dL) | Age (years) | -0.0446306 | -3.775233 | 3.685971 | 0.904 |
|  | Male (%) | 2.168221 | -126.3092 | 130.6457 | 0.866 |
|  | BMI (kg/m^2^) | -0.5572983 | -13.1976 | 12.083 | 0.675 |
|  | HbA1c (%) | 3.880055 | -97.98005 | 105.7402 | 0.713 |
|  | Duration of diabetes (years) | 0.0020102 | -1.526747 | 1.530767 | 0.989 |
| SAEs | Age (years) | 0.0422763 | -0.0929831 | 0.1775358 | 0.434 |
|  | Male (%) | 1.613631 | -6.584357 | 9.811618 | 0.614 |
|  | BMI (kg/m^2^) | -0.1206634 | -0.417878 | 0.1765512 | 0.323 |
|  | HbA1c (%) | -0.5281924 | -1.65285 | 0.5964654 | 0.262 |
|  | Duration of diabetes (years) | -0.0096111 | -0.1428883 | 0.123666 | 0.851 |
